# Innovative psycho-educational program to prevent common postpartum mental disorders in primiparous women: a before and after controlled study

**DOI:** 10.1186/1471-2458-10-432

**Published:** 2010-07-23

**Authors:** Jane RW Fisher, Karen H Wynter, Heather J Rowe

**Affiliations:** 1 Centre for Women's Health, Gender and Society, Melbourne School of Population Health, University of Melbourne, Victoria 3010, Australia

## Abstract

**Background:**

Universal interventions to prevent postnatal mental disorders in women have had limited success, perhaps because they were insufficiently theorised, not gender-informed and overlooked relevant risk factors. This study aimed to determine whether an innovative brief psycho-educational program for mothers, fathers and first newborns, which addressed salient learning needs about infant behaviour management and adjustment tasks in the intimate partner relationship, prevented postpartum mental health problems in primiparous women.

**Methods:**

A before and after controlled study was conducted in primary care in seven local government areas in Victoria, Australia. English-speaking couples with one-week old infants were invited consecutively to participate by the maternal and child health nurse at the universal first home visit. Two groups were recruited and followed sequentially: both completed telephone interviews at four weeks and six months postpartum and received standard health care. Intervention group participants were also invited to attend a half-day program with up to five couples and one month old infants, facilitated by trained, supervised nurses. The main outcome was any Composite International Diagnostic Interview (CIDI) diagnosis of Depression or Anxiety or Adjustment Disorder with Depressed Mood, Anxiety, or Mixed Anxiety and Depressed Mood in the first six months postpartum. Factors associated with the outcome were established by logistic regression controlling for potential confounders and analysis was by intention to treat.

**Results:**

In total 399/646 (62%) women were recruited; 210 received only standard care and 189 were also offered the intervention; 364 (91%) were retained at follow up six months postpartum. In women without a psychiatric history (232/364; 64%), 36/125 (29%) were diagnosed with Depression or Anxiety or Adjustment Disorder with Depressed Mood, Anxiety, or Mixed Anxiety and Depressed Mood in the control group, compared with 16/107 (15%) in the intervention group. In those without a psychiatric history, the adjusted odds ratio for diagnosis of a common postpartum mental disorder was 0.43 (95% CI 0.21, 0.89) in the intervention group compared to the control group.

**Conclusions:**

A universal, brief psycho-educational group program for English-speaking first time parents and babies in primary care reduces *de novo *postpartum mental disorders in women. A universal approach supplemented by an additional program may improve effectiveness for women with a psychiatric history.

**Trial registration:**

ACTRN 12605000567628.

## Background

Postnatal mental health problems in women are associated with disability, reduced social participation and diminished caretaking capacity [1] and constitute a significant public health problem [2], which has proved difficult to prevent [3].

### Nature and prevalence of postpartum mental health problems

The predominant focus of research, policy initiatives, clinical practice recommendations and health education has been postnatal depression, but there is increasing evidence that postnatal anxiety disorders are at least as common, but less well recognised than depression [4,5]. Brockington [1] in a review of postnatal psychiatric disorders concludes that women identified through screening as depressed actually have heterogeneous conditions including posttraumatic stress disorder, panic, phobic, obsessional and generalised anxiety disorders, adjustment disorders and depression. These are situation-focused, disabling and often reflect adversity [6]. Even among those who meet diagnostic criteria for major depression, severity ranges from mild to severe and most depression after childbirth is minor and not major [7]. Brockington [1] argues that 'postnatal depression' therefore has value as a lay term, but is imprecise as a clinical or a research construct.

This lack of clarity is reflected in widely divergent estimates of prevalence for probable depression as assessed by the Edinburgh Postnatal Depression Scale [8] in women in high income countries from 3.7% to 36% [9] and for diagnoses of postnatal generalised anxiety disorder from 4.4 to 8.5% [10]. There is less evidence about the prevalence of panic disorders in women after childbirth, but a much higher de novo onset of panic attacks in the first twelve weeks postpartum (10.9%) than expected by chance (0.92%) is reported [10]. In women with a history of panic attacks, symptoms tend to increase after childbirth, but not during pregnancy [10]. The prevalence of adjustment disorders arising in response to the birth of a baby has not been established. However, there is recognition that a proportion of mothers-of-infants who seek help for early parenting difficulties do not meet diagnostic criteria for depression or an anxiety disorder, but do have higher than population average scores on the Edinburgh Postnatal Depression Scale (EPDS) [8]. An expanded conceptualisation, including adjustment disorders, is required to recognise their needs [11].

### Universal interventions to prevent postnatal depression

In addition to treatment for people with current mental health problems, a comprehensive approach to mental health service delivery must include mental health promotion and the prevention of mental disorders [12]. A range of interventions to prevent postnatal mental health problems, principally 'postnatal depression', have been tested in randomised controlled trials. These have included secondary prevention via indicated interventions for women with current clinically significant depressive symptoms, selective interventions for women identified by screening as at risk of developing depression and universal interventions offered to all women to reduce population prevalence [13-17]. Universal strategies are preferred; because even small reductions in population prevalence have a greater public health benefit than treating individuals who are already symptomatic [18]. They are also less stigmatizing and more likely to be used [19]. Systematic reviews have concluded that screening measures administered in pregnancy have low positive predictive values, probably because events after childbirth are more salient determinants of postnatal depression [15].

There have been seven trials of universal postnatal interventions offered to unselected populations of women who have recently given birth. Five tested strategies for use with individual women: debriefing with a psychologist about childbirth experiences [20] or a midwife listening visit during the postnatal hospital stay [21]; earlier-than-usual postnatal visit to a primary care physician [22]; ten three-hour home visits involving practical assistance and emotional care from a specifically trained support worker in the first postpartum month [23]; and an information pack containing specific, salient written information about maternal health, sleep and support needs and management of infant crying, with or without an invitation to attend a facilitated new mothers' group [24]. The other two trials assessed comprehensive community-based interventions which involved increasing the skills of primary health care nurses to identify women's physical and mental health conditions and initiate referral to appropriate health services [25,26]. Lumley et al [26] also provided specific training for general practitioners and community development aimed at enhancing local facilities and services for parents of newborns.

Of these varied strategies, only Lavender et al's midwife-listening visit [21] and MacArthur et al's primary health care intervention [25] were associated with reduced mental health problems in the treated compared to the control group. In Lavender et al's study, there was apparent selection bias in that 60% of participants were single women and the very high rates of self-reported depression and anxiety in the control arm (50% classified as having clinically significant symptoms) led reviewers to conclude that it is a 'true outlier' [27]. McArthur et al's intervention [25] was embedded in the UK National Health Scheme in which women are more likely to have an established relationship with a primary health care practice than in other health systems. All studies were adequately powered, analyzed by intention to treat, had properly concealed random allocation to trial arms and blinded assessment of outcomes. There were some methodological limitations: attrition greater than 20% at final assessment [22-25] and in one trial there was poor compliance with the intervention [24]. Dennis [3] concludes that these studies are generally of good methodological quality. The findings raise questions about why most of the interventions were not effective. A number of explanations emerge.

First, most conceptualised postnatal mental health problems as depression and/or general mental health morbidity and role functioning assessed by the SF-36 [28]. Anxiety was an outcome in only two trials. Lavender [21] reported reduced anxiety symptoms, but Priest [20] found no differences between groups in acute stress disorders. It is possible therefore that the interventions might have had undetected benefits for mental health problems other than depression or acute stress disorders.

Second, none of the trials analysed outcomes by prior psychiatric history, so it is also possible that the interventions had differential effects in women with and without a history of mental health problems, which were not detected [27,29].

Third, the mechanisms by which the interventions were proposed to reduce mental health morbidity did not target modifiable risk factors directly. Of the four well-established risk factors for depression after childbirth: personal psychiatric history, coincidental adverse life events, quality of relationship with the intimate partner and insufficient social support [30], the latter two are the most readily modified in the postpartum period. Most of the universal interventions addressed low social support, but through the provision of enhanced professional care outside the domestic sphere particularly in primary health care consultations, and not by seeking to improve the quality of a woman's intimate relationships [20,21,23-26,29].

### New avenues for prevention

#### Intimate partner relationship

There is consistent evidence that the quality of relationship with the intimate partner is associated with postnatal mental health in women. It has been found to act both protectively and to increase risk. Women, who experience their partners as welcoming the pregnancy, and providing empathic support and encouragement, have better mood [31-33]. In contrast, women, who feel unable to confide in their partners or are experiencing conflict, poor communication or dissatisfaction in the relationship have worse mood [31,34-42]. Although the evidence is consistent, few investigators have operationalised how difficulties in the intimate partner relationship are enacted in day-to-day behaviours. Only two randomised controlled trials of universal interventions for the prevention of postnatal mental health problems, both offered during pregnancy in North America, included partners. Fifty years ago Gordon et al [43] included men in two additional childbirth education classes, not only modelling that the work of parenting is a shared obligation, but also providing guidance to assist men to be sensitised to the demands of this life change for women. There were significantly fewer 'emotional upsets' in women in the intervention than the standard care group six months postpartum. Midmer [44] tested the effects of two additional 3-hour classes which focused on increasing: couples' appreciation of potential feelings of isolation, ambivalence, conflict, resentment and guilt in new mothers; and skills for managing relationships with extended family, a fretful baby, and the redistribution of household chores, using role-play and practice in problem solving and communication techniques in a standard childbirth education program for women and men. There was lower anxiety in women and men in the intervention than in the standard care group six weeks and six months postpartum. Gordon et al used non-standardised outcome assessments and neither study controlled for cluster effects, but Gordon et al's study is cited as evidence of the importance of including women's partners in strategies to promote postpartum mental health [27].

#### Unsettled infant behaviour

To date, most investigations in this field have presumed that infants' behaviour reflects parenting factors [45], in particular that prolonged infant crying is a consequence of maternal depression [46]. Few have acknowledged that the relationship might be reciprocal and that infant behaviour might exert an adverse effect on a mother's confidence and affect. Infant behaviour, especially prolonged inconsolable crying, frequent night-time waking, short daytime sleeps and feeding difficulties are very common reasons for mothers of infants to seek help [47,48]. Mothers of infants who cry excessively report significantly more parenting stress and less sense of competence and efficacy than other mothers, and do not experience their infants as a source of positive reinforcement [49].

#### Occupational fatigue

Profound fatigue is widespread among mothers of newborns but is often normalised or trivialised, despite the adverse impact it exerts on normal daily functioning [50]. It has been regarded as symptomatic of depression, but an alternative view is that it arises because the unpaid workload of mothering a newborn is severely underestimated [51]. Poor infant sleep and maternal fatigue have been shown in a prospective investigation to precede the onset of depressive symptoms in women [52]

#### Social theory of depression

Brown and Harris's [53] social theory proposes that depression in women arises from experiences of entrapment and humiliation, which we argue are salient to the circumstance of mothering a newborn. The work of infant care is intrinsically confining. If the baby is responsive and rewards the mother by quieting to her soothing, smiling, interacting, suckling easily, and developing along at least an average trajectory, the baby provides gratification. In contrast, an infant who resists soothing, cries inconsolably, or is difficult to breastfeed can be experienced as critical and unappreciative. The work of mothering an infant and managing a household in which an infant lives is repetitive, isolated, never complete, and can be ungratifying. A mother of a newborn depends on her partner for recognition and affirmation of her endeavours and is especially vulnerable to his criticism, which can be humiliating. At this life phase women have increased dependence on intimate relationships, and reduced interactions with workplaces and the broader community.

None of the universal postnatal interventions included partners or attempted to modify day-to-day interactions in this relationship, included infants and addressed infant behaviour, or attempted to prevent occupational fatigue. We postulate that depression and anxiety in mothers of newborns can be conceptualised as reflecting poorly functioning intimate relationships, which are potentially modifiable mediated by fatigue.

The aim of this study was to assess whether *What Were We Thinking! *(*WWWT*) a brief, novel, highly structured, universal psycho-educational intervention for mothers, fathers and a first newborn, which addresses the intimate partner relationship, infant behaviour management, and thereby the mediating effects of occupational fatigue (see [29] for a detailed description) is effective in reducing the common maternal mental health problems of Depression or Anxiety or Adjustment Disorder with Depressed Mood, Anxiety, or Mixed Anxiety and Depressed Mood.

## Methods

### Study design

The *What Were We Thinking! *intervention was designed to be highly diffusible amongst families and social networks and includes attractive take home materials for ongoing reference. To prevent contamination of the standard care group with the intervention, we used a before and after controlled study design [54]. We first recruited and followed a control group who received standard primary postnatal care. Immediately after this, a second group was recruited and followed in the same way, but in addition to standard postnatal care, this group was invited to attend the intervention. Outcomes in the two groups were compared, controlling for baseline differences.

### Setting

The study was conducted in seven local government areas (LGAs) in the Australian state of Victoria (population 5.2 million [55]). Diverse LGAs were selected by Socio-Economic Indices for Areas (SEIFA) to represent a range of areas across the spectrum of socioeconomic advantage and disadvantage [56]; three were from rural Victoria and four were in metropolitan Melbourne.

Recruitment to the control group took place from February to December 2006, and to the intervention group from February to December 2007. The intervention was conducted when babies were approximately four weeks old in easily accessed Maternal and Child Health Centres in the participating local government areas. Six-month follow-up of control group participants was completed in June 2007 and of the intervention group in June 2008.

### Participants

All couples with healthy firstborn infants, sufficient English language proficiency to complete telephone interviews, both partners of which were willing to participate in the study and aged over 18 years, were eligible. Maternal and child health nurses provided verbal and written information about the study at the universal home visit and in the postnatal ward of a private maternity hospital. Interested couples provided contact details and were telephoned by research staff within one week. Women and men who agreed to participate returned individual signed consent forms by mail.

### The intervention program

*What Were We Thinking! *(*WWWT*) is a highly structured, interactive, gender-informed, couples-based, psycho-educational program for parents and a first newborn to promote confident parental caretaking, optimise functioning in the intimate partner relationship, improve infant manageability and reduce common postnatal mental disorders in women [29].

#### Theoretical principles of the intervention

The theoretical principles of the *WWWT *program are, first, that postpartum anxiety is as important as depression and requires explicit attention; however, as depression and anxiety are not easily distinguished, they are addressed most effectively together. Second, that partner and infant behaviours can be modified to decrease those that contribute to maternal depression and anxiety and increase those that promote maternal confidence and sense of competence. Third, that women desire care from and gratification within these relationships and not increased care from health professionals. Fourth, that improvements in on-going day-to-day interactions are of fundamental importance to mental health promotion. Fifth, that this knowledge needs to be made available at a critical developmental stage and in a readily comprehensible form. Finally, that the language of the intervention is crucial and needs to challenge gender stereotypes and honour the work of mothering newborns.

#### Psycho-educational approach

*WWWT *is psycho-educational in addressing theoretically plausible psychological mechanisms using an educational approach to meet parents' learning needs. The program aims to: minimise experiences of humiliation through increasing fathers' understanding and empathy; counter experiences of entrapment by promoting infant care as a shared endeavour in which parents with comparable competence can permit each other independent or shared leisure [53]; and promote cognitive- rather than emotion-focused responses to infant crying by building skills to respond in non-avoidant ways. Together these strategies are expected to promote gratifying and rewarding intimate interactions rather than frustrating and diminishing ones, minimise maternal fatigue and thereby lead to increased parental confidence; more settled infant behaviour; and reduced depression, anxiety and adjustment disorders [29].

The educational approach addresses the provision of salient knowledge and opportunities to learn new skills. These are difficult to acquire through self-learning at this life stage because of fatigue, and because it is difficult for most people to distinguish between resources that are evidence-based, and those that constitute personal experience or opinion. Principles of adult learning are used and include group discussion, focused tasks to be undertaken individually and then discussed as a couple; practice in problem solving and negotiation, hands-on supported practice in infant settling, short talks and practical demonstrations. Anxiety is contained by a supportive, non-judgemental and knowledgeable facilitator. Very careful language-use is prescribed so that gender stereotypes are challenged, fathering and mothering are positioned as different but of equal importance and emotions are named and normalised without the use of psychiatric labelling.

#### Specific content

*WWWT *has 13 sections, grouped into two components: "About Babies" and "About Mothers and Fathers". About Babies includes sections about infant temperament, crying and fussing, recognition of tired cues, sleep needs, establishing feed-play-sleep routines of daily care and sustainable settling strategies. Opportunities to practise wrapping their babies and settling them to sleep are provided in the session. About Mothers and Fathers includes sections about differences between how parenthood had been imagined and reality; recalling the difficult and pleasing aspects of the baby's birth; recognising, naming and renegotiating the unpaid workload; acknowledging the losses and gains of parenthood; thinking about experiences within their families of origin that they wish to duplicate or to leave behind; and identifying gaps in support. This component provides language and strategies to assist couples to understand and respond effectively to changed needs for support, re-negotiate the paid and unpaid household workload fairly and manage the losses and gains associated with becoming parents. A folder containing a short book covering program content in accessible plain language and illustrations, and worksheets for each section is used during the program and taken home by couples for later reference.

#### Program delivery

Antenatal education for women and their partners is well established and there are high participation rates in Australia, but it does not continue postpartum when parents have high learning needs. Programs were held on Saturday mornings to maximise fathers' participation with groups of up to five couples and their babies. Materials were sent by mail to those who did not attend the face-to-face intervention.

#### Program fidelity

Program facilitators were three maternal and child health nurses, experienced in leading groups, who had attended a half day training session conducted by JF (clinical psychologist) and HR (adult educator). Training included didactic sessions to address relevant theory, role play to promote use of appropriate language to describe household work and challenge gendered stereotypes, and practice in supporting parents in infant settling techniques. The program was pilot tested with eleven couples and their infants and feedback was incorporated prior to implementation.

Fidelity to the program was upheld by adherence to the Facilitators' Handbook containing program theory, learning outcomes, group strategies and interactive worksheets for each section. Telephone and email communication with lead investigators was available to facilitators for an immediate response to unanticipated problems. The research coordinator provided informal weekly supervision and support, and formal supervision took place in face-to-face settings with JF and HR at bi-monthly intervals throughout the implementation phase.

### Standard care

Participants in the control group received usual primary health care.

### Data sources

Data were collected by computer-assisted-telephone-interviews (CATIs) conducted at approximately two weeks (baseline interview) and six months (follow up interview) postpartum.

*The primary outcome *was any diagnosis of a disorder meeting DSM IV criteria [57] for a Specific or Social Phobia, Panic with or without Agoraphobia, Generalized Anxiety Disorder, Dysthymia, Major or Minor Depressive Episode in the first six months postpartum as assessed by the relevant module of the Composite International Diagnostic Interview (CIDI) [58]. Adjustment disorders were diagnosed as the DSM IV criteria of feeling low and sad most of the day, nearly every day for at least two weeks since the birth of the baby and that it had not followed bereavement (Adjustment Disorder with Depressed Mood); a period of at least a month since the baby's birth of feeling worried, tense or anxious about everyday problems such as work, family or life with the baby (Adjustment Disorder with Anxiety), or both (Adjustment Disorder with Mixed Anxiety and Depressed Mood). These diagnoses were not made if the participant's other symptoms met criteria for Major or Minor Depressive Episode or Generalised Anxiety Disorder.

*Psychiatric history *was assessed by both study-specific questions and the CIDI as self reported lifetime history of treatment for alcohol or drug dependence, depression, eating disorder, or symptoms meeting criteria for panic attack in non life-threatening situations.

*Potential confounders *were assessed using study-specific questions at the baseline interview. Maternal factors included: age, country of birth, language spoken at home, marital, educational and occupational status, self-rated health, gravidity, appraisal of partner support and self-rated confidence on discharge from maternity hospital. Infant factors included: multiple birth, sex, birthweight, gestation at birth, age, health status and method of feeding. Standardised psychometric instruments were used to assess personality; depressive symptoms, infant behaviour and quality of relationship with the intimate partner (see Table 1).

**Table 1:** Standardised instruments and their psychometric properties

Variable	Instrument	Scale description	Psychometric properties
Primary outcome			

Common mental disorders	Composite International Diagnostic Interview (CIDI) [58]	Widely used, completely structured lay-administered clinical interview that yields DSM-IV and ICD-10 diagnoses through algorithms.	Concordance between CIDI diagnoses and Structured Clinical Interview for DSM-IV (SCID) diagnoses of depression (κ = 0.54) and anxiety disorders (κ = 0.48) [70].

Baseline factors			

Personality factors which might increase vulnerability to mental health problems	Vulnerable Personality Style Questionnaire (VPSQ)	Vulnerability Subscale measures over-sensitivity to the opinions of others and lack of assertiveness Range of scores 6 to 30	Cronbach's α for internal consistency 0.77; test - retest reliability 0.82 p < 0.01, in a model predicting postnatal depression sensitivity 0.14 and specificity 0.94 [71]; correlation with self-esteem 0.58 [72].

Depressive symptoms	Edinburgh Postnatal Depression Scale (EPDS) [8]	10-item self- report scale to screen for probable depression during the postnatal year in research and health care settings. Range of scores 0 to 30	Standardised α 0.87; sensitivity 0.85 and specificity 0.77; positive predictive value 0.83[8].

Quality of relationship with intimate partner	Intimate Bonds Measure (IBM) Subscales: Care, Control	Care subscale assesses sensitivity, warmth, emotional responsiveness, trust, physical gentleness and kindness. Control subscale assesses coercion, dominance, exertion of power and extent of criticism. Range of scores 0 to 36 for each subscale	Care: Cronbach's α 0.94; correlation with clinical interview ratings of quality of relationship 0.68. Control: Cronbach's α 0.89 and correlation with clinical interview ratings of quality of relationship 0.74 [73].

Duration and frequency of infant crying and fussing in a 24 hour period	Barr Chart [74] Possible range 0 to 24 hours	Parental diary of duration of episodes of crying, fussing, sleeping, and content infant behaviours.	Reliably completed by parents, high correlation with tape recordings: for frequency (r = 0.85, p = 0.002) and duration (r = 0.90, p = 0.001) of episodes [74].

*Fidelity of intervention *delivery was assessed by standard program evaluation checklists, completed by facilitators after each implementation. Facilitators rated how well the objectives of each of the 13 individual components of the program had been achieved, using a scale of 1 to 5 (1 = not at all; 5 = completely), and recorded details of unforeseen events that influenced delivery of the intervention.

*Potential effect modifiers *occurring between interviews were assessed at the follow up interview including: self-reported adverse life events, which were rated according to number and severity of events [59], and use of mental health or early parenting services.

### Procedure

Interviews were conducted uniformly by trained telephone interviewers who had no other involvement in the study. Repeat contact attempts were made for up to one month at the preferred time that had been nominated at recruitment to participants who were unavailable. Attrition was minimised by the use of participant-provided additional contact telephone numbers. An AUD 25 shopping voucher to compensate for inconvenience was posted to participants who completed all interviews. CATIs were identical for intervention and control participants with the exception of specific questions in the follow up interview about the intervention program for participants in the intervention group.

### Sample size

The intervention program was designed for groups of five couples. The sample size calculation included a correction to adjust for any correlation between responses within the same group. Assuming an intraclass correlation of 0.1 and an average cluster size of 5, the inflation factor for the intervention group was 40%. Thus the required ratio of control group participants to intervention group participants was 1:1.4. For a change of 10% in the prevalence of common mental disorders in women in the first six months postpartum, a two group continuity corrected chi-sq test with a 0.05 two-sided significance level will have 80% power to detect the difference between a control group proportion of 0.2 (20%) and a *WWWT *program group proportion of 0.1 (10%) (OR 0.444), with groups of 193 and 246, respectively, a total sample size of 439.

### Ethics

Approval to conduct the study was provided by the Department of Human Services Victoria Human Research Ethics Committee and the University of Melbourne's Human Research Ethics Committee.

### Data management and analysis

Maternal age and psychometric measures of maternal mood, personality and infant crying and fussing were continuous variables. Binary variables were constructed for: occupation, which was coded by the Australian Standard Classification of Occupations (ASCO 1 to 4 and ASCO 5 - 8) [60]; whether English or another language was spoken at home; any or no psychiatric history; primi- or multigravid; unexpected or intended pregnancy; self rating as confident or anxious on discharge from maternity hospital; any breastfeeding or formula feeding and sufficient or insufficient help and support from partner at baseline. Study group was intervention or control and primary outcome was presence or absence of depression, or anxiety or adjustment disorders in the first six months postpartum.

Baseline characteristics of those retained in the study were compared to those lost to follow-up. Univariate analyses were used to establish all significant differences in baseline variables between control and intervention groups, and to test associations between baseline variables and the primary outcome. Chi-square tests were used for binary variables; Mann-Whitney tests for ranked and ordered categorical variables, and t-tests for continuous variables. Statistical significance was set as p < 0.05. Between group differences are presented as means (95% confidence intervals) for continuous variables, and proportions (95% confidence intervals) for categorical variables

The relationship between participation in the intervention and the outcome was tested by logistic regression in STATA [61], adjusting for potential confounders selected a priori from the univariate analyses. Variance Components Estimation was specified in the model and robust standard errors were used to adjust for clustering of individuals attending the same intervention program. Analysis was by intention to treat. Univariate analysis confirmed that the relationship between study group and the primary outcome was mediated by psychiatric history. An interaction term for psychiatric history and study group was therefore included in the model.

The model was adjusted for variables that differed between groups at baseline and for those associated independently with the primary outcome. All baseline data from a small set of cases were lost owing to server failure. Under an assumption of Missing Completely at Random, we used complete case analysis, thus excluding these cases and an additional ten cases with a small amount of missing data. Results of the model are presented as adjusted odds ratios and 95% confidence intervals and according to the TREND guidelines [62]. As a sensitivity analysis, we re-ran the model on the same number of cases, but excluding from the analysis the variable with the most missing cases. The odds ratios in this analysis were compared with the results of the original analysis.

## Results

### Participants

**Figure 1:**
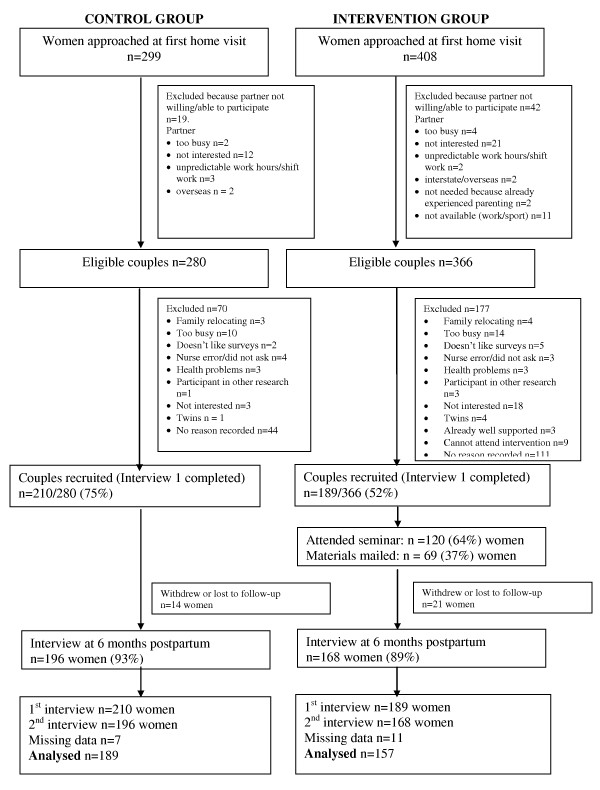
**Flow chart of recruitment and participation**.

Of the 646 eligible couples invited to participate, 399 women completed the first interview giving an overall recruitment fraction of 61.8%. Of these, 364 (91.2%) women completed the follow up interview. Women who could not be contacted by telephone for the follow up interview (n = 35) had significantly lower educational attainment, higher self-rated confidence on discharge from maternity hospital and reported fewer breastfeeding problems at the baseline interview than those who had completed both interviews (n = 364). Server failure led to loss of baseline data from eight participants and data for at least one baseline variable in the final model was missing for eighteen participants (5%). These were excluded, leaving 346 cases with complete data in the final model (Figure 1).

The baseline interview was conducted at mean (SD) 4.1 (2.3) weeks and the follow up interview at 27.6 (5.5) weeks postpartum. There were no significant between-group differences in infant age at either interview. Women in the intervention group were significantly older, more likely to speak English at home, to have completed post-secondary education, to be in professional or managerial employment and to be multigravid, and less likely to report that the index pregnancy had been unintended than women in the control group. Babies in the intervention group were more likely to be breastfed and cried and fussed for longer periods than those in the control group (see Table 2).

**Table 2:** Baseline characteristics of participants by study group

					95% CI for the difference	
		Control (n = 210)	Intervention (n = 189)	p	Lower	Upper
**Sociodemographic characteristics**						

Age	Mean (s.d.)	30.2 (5.31)	31.62 (4.79)	0.004	-2.47	-0.47

Aboriginal/Torres Strait Islander	n (%)	2 (1)	1 (0.5)	0.625		
		
English spoken at home	n (%)	198 (94.3)	186 (98.4)	0.03		
		
Married	n (%)	148 (70.5)	135 (71.4)	0.834		
		
Completed post-secondary education	n (%)	135 (64.3)	157 (83.1)	<.001		
		
Employment ASCO classification *:						
1 and 2	n (%)	78 (37.3)	111 (59.0)	<. 001		
			
3 and 4	n (%)	35 (16.7)	31 (16.5)			
			
5 and 6	n (%)	73 (34.8)	34 (18.1)			
			
7, 8 and 9	n (%)	24 (11.5)	12 (6.4)			

**Health and reproductive history**						
Self-rated health good or excellent	n (%)	184 (87.7)	177 (93.6)	0.321		
		
Psychiatric history (panic episodes only)	n (%)	58 (27.6)	42 (22.2)	0.401		
		
Psychiatric history (other disorders)	n (%)	16 (7.6)	25 (13.2)	0.065		
		
More than one previous pregnancy	n (%)	40 (19.1)	53 (28)	0.036		
		
Unexpected pregnancy	n (%)	56 (26.8)	29 (15.3)	0.005		
		
Conceived by ART^+^	n (%)	18 (8.6)	17 (9)	0.893		
		
Caesarean birth	n (%)	69 (32.9)	59 (31.2)	0.726		

**Psychological factors**						
		
Confident on leaving hospital	n (%)	123 (58.9)	91 (50.0)	0.079		
		
Can confide in partner	n (%)	185 (88.5)	163 (89.6)	0.742		
		
Feels supported by partner	n (%)	200 (96.2)	173 (95.1)	0.596		
		
Vulnerable personality style (VPS vulnerability subscale score)	Mean (s.d.)	12.75 (4.16)	12.7 (3.96)	0.904	-0.75	0.85

Current mood (EPDS)	Mean (s.d.)	5.81 (4.01)	6 (3.75)	0.646	-0.96	0.59

Partner relationship (IBM Care subscale score)	Mean (s.d.)	33.34 (3.76)	32.76 (4.12)	0.149	-0.21	1.36

Partner relationship (IBM Control subscale score)	Mean (s.d.)	4.58 (4.26)	4.76 (4.75)	0.694	-1.08	0.72

**Infants**						

Twins	n (%)	3 (1.4)	1 (0.5)	0.368		
		
Female	n (%)	109 (52.7)	92 (48.9)	0.46		
		
Birth weight (g)	Mean (s.d.)	3503.6 (437.5)	3406.9 (510.4)	0.054	-1.76	195.30

Gestation at birth (weeks)	Mean (s.d.)	39.86 (1.45)	39.62 (1.56)	0.116	-0.06	0.54

Infant age at interview (weeks)	Mean (sd)	4.3 (2.5)	3.9 (2.2)	0.19	-0.17	0.81

Rating of baby's health Good or Excellent	n (%)	201 (96.2)	174 (95.6)	0.401		
		
Currently breastfeeding	n (%)	163 (78)	160 (87.9)	0.01		
		
Had breastfeeding problems	n (%)	50 (23.8)	46 (24.3)	0.902		
		
Length of time baby cried or fussed in previous 24 hours (hours)	Mean (s.d.)	2.86 (2.04)	3.52 (2.04)	0.005	-1.11	-0.2

* ASCO Australian Standard Classification of Employment classification [60]

1 and 2: Managers, Administrators, Professionals

3 and 4: Associate Professionals, Tradespersons, Associated Workers

5 and 6: Advanced/Intermediate Clerical, Sales, Service Workers

7, 8 and 9: Intermediate Production and Transport Workers, Elementary Clerical, Sales and Service Workers, Labourers & Related Workers, students & unemployed

^+ ^ART assisted reproductive technology

### Intervention program fidelity, participation and satisfaction

A total of 37 intervention programs were implemented with women, their partners and babies, with a mean (SD) group size of 2.7 (1.6) families, at mean (SD) 6.6 (2.5) weeks postpartum. Facilitator evaluation checklists, which were available for 36/37 (97%) programs, showed that in each program all the individual sections had been delivered, and that the objectives of the individual sections were achieved (rated at least 4/5) in almost all of these (491/504; 97%).

A total of 120/189 (63.5%) of women in the intervention group attended the program and received a folder of written materials for take-home reference. Most of those who booked and confirmed, but did not attend a program, did not provide a reason, but those who did, cited unexpected illness or partner work or sporting commitments. The folder of written materials was posted to all those who did not attend in person. Most of the women (54/61; 89%) who were sent materials by mail reported at the follow up interview that they had received them.

Anonymous participant evaluation questionnaires completed by 98/120 (82%) women at the end of the intervention program revealed that 92 (94%) reported increased understanding of infant sleep needs, 81 (83%) increased understanding of infant temperament, 91 (93%) increased understanding of infant sleep and settling strategies, 71 (72%) could now talk more effectively about parenting with their partners and 64 (66%) already reported increased confidence in infant care.

### Mental health outcomes

The primary outcome was a CIDI diagnosis of Depression or Anxiety or Adjustment Disorder with Depressed Mood, Anxiety, or Mixed Anxiety and Depressed Mood in the first six months postpartum (Table 3). Of the 117 women diagnosed as having experienced a disorder, 52 (44.4%) had no psychiatric history and were classified as having a *de novo *condition. The remainder (65/117; 55.5%) had histories of depression, panic, eating or substance abuse disorders and were classified as having a recurrent mental health problem.

**Table 3:** Women meeting diagnostic criteria for common mental disorders in the first six months postpartum by psychiatric history and study group (n = 364)

	Whole sample (n = 364)			Without psychiatric history n = 232 (63.7%)		With psychiatric history n = 132 (36.3%)	
**Disorder**	**Control** **n = 196**	**Intervention** **n = 168**	**Total** **n = 364**	**Control** **n = 125**	**Intervention** **n = 107**	**Control** **n = 71**	**Intervention** **n = 61**

None	129	118	247	89	91	40	27

Adjustment disorder with anxious mood	36	28	64	22	12	14	16

Adjustment disorder with depressed mood	4	2	6	2	1	2	1

Adjustment disorder with mixed mood	6	6	12	1	3	5	3

Dysthymia	0	2	2	0	0	0	2

Anxiety disorder	20	12	32	10	0	10	12

Depression	1	0	1	1	0	0	0

Total disorders n (%)	67 (34.2)	50 (29.8)	117 (32.1)	36 (28.8)	16 (15.0)	31 (43.7%)	34 (55.7%)

In the group without a psychiatric history, the absolute risk reduction associated with the intervention was 0.14 (14%), and the relative risk reduction was 0.48 (48%). The original effect size on which our power calculation was based was conservative (10% difference); we showed a larger-than-postulated effect size, which reached significance in our smaller-than-necessary sample size.

### Factors associated with mental health outcomes at 6 months

Use of mental health and residential early parenting services since the birth were potential effect modifiers as they had been used by significantly more participants in the intervention than in the control group. Univariate analysis revealed that use of both these services was associated with psychiatric history. Specifically, 69% of those who consulted a mental health practitioner (p < 0.001) and 83% (p < 0.01) of those who attended a residential early parenting service had a psychiatric history. There were no significant differences between study groups in the use of these services by women without a psychiatric history and therefore these were not included in the model. There were no significant between-group differences in number or severity of coincidental adverse events experienced between baseline and follow up.

In the final model, adjusting for all other factors, three variables remained significant predictors of the primary outcome: EPDS score at baseline interview, study group, and the interaction term for psychiatric history and study group, indicating that the effect of the intervention varied by whether or not respondents reported a psychiatric history (see Table 4).

**Table 4:** Factors associated with diagnosis of common mental disorders at outcome (n = 346)

Variable	Reference Category	Odds Ratio	Robust Standard. Error	p	**95% CI**.	
						

**Respondent age**		0.97	0.03	0.31	0.90	1.03

**Language at home**	English	1				

	Other than English	0.88	0.65	0.87	0.21	3.78

**Occupation**	Skilled	1				

	Unskilled	0.81	0.24	0.47	0.44	1.44

**Number of pregnancies**	First	1				

	More than one	1.23	0.38	0.50	0.67	2.26

**Unexpected pregnancy**	No	1				

	Yes	1.28	0.42	0.45	0.67	2.45

**EPDS total score**		1.14	0.05	0.00	1.05	1.24

**VPS vulnerability score**		1.07	0.04	0.06	1.00	1.16

**Support from partner**	Yes	1				

	No	2.57	1.63	0.14	0.75	8.88

**Breastfeeding**	Yes	1				

	No	1.19	0.40	0.61	0.61	2.31

**Confidence at discharge from maternity hospital**	Yes	1				

	No	1.10	0.30	0.74	0.64	1.88

**Time baby cried or fussed in 24 hours**		0.96	0.07	0.62	0.83	1.11

**Psychiatric history**	No	1				

	Yes	1.59	0.54	0.17	0.82	3.09

**Study group**	Control	1				

	Intervention	0.43	0.16	0.02	0.21	0.89

**Study group × psychiatric history**	*Interaction term*	4.27	2.21	0.01	1.53	11.78

For participants with no psychiatric history, being in the intervention group was associated with a significantly reduced odds (OR 0.43; 95% CI 0.21, 0.89; p = 0.022) of a diagnosis of a mental disorder. A linear combination of estimates was used to calculate the odds ratio associated with diagnosis of a mental disorder for participants with a psychiatric history in the intervention group (OR = 1.8; 95% CI 0.92, 3.71; p = 0.082) and demonstrated that being in the intervention group was not associated with significantly increased odds, compared to the control group, and thus the intervention did not cause harm.

### Sensitivity analysis

The model was re-run on data from these same 346 respondents, excluding as predictor the number of hours the infant had cried or fussed in the past 24 hours, which had the highest number of missing values and was not a significant predictor. None of the odds ratios changed by more than 5%. The model was therefore re-run on all cases for which all the remaining predictors were available (n = 353) and the results were not different from those already reported.

## Discussion

This before and after controlled study provides the first evidence that a brief, structured, universal, salient, gender-informed psycho-educational intervention offered in local settings within the first month postpartum appears to be effective in reducing the onset of the common postpartum mental health problems of Depression or Anxiety or Adjustment Disorder with Depressed Mood, Anxiety, or Mixed Anxiety and Depressed Mood in partnered mothers of a first infant who have no history of psychiatric illness.

There are insufficient comprehensive data to establish current and lifetime prevalence estimates of DSM IV Axis I disorders, including, anxiety and adjustment disorders in women in the first six months after childbirth. However, the lifetime prevalence (36%) and 6-month prevalence (32%) reported in this study are consistent with available evidence. A systematic investigation of 1066 women attending for routine antenatal care in Pisa, Italy reported that lifetime prevalence was 50.4% and that 26.3% met criteria for current disorders [63]. In Australia Hiscock and Wake [64] found in a systematic community-based survey of 738 mothers of seven- month-old infants that 15% scored more than 12 and another 18% scored 10 - 12 on the EPDS.

It is well established that evaluation of complex health promotion interventions in real world settings is challenging [65]. In accordance with the criteria for evaluation of before and after controlled studies [66], we argue that the findings of this study are important, of notable magnitude and that relevant determinants were not ignored; participants in the first and second group met identical eligibility criteria and were recruited systematically from the general population of primiparous mothers and there was no co-occurring service change that might have contributed to a general trend in improvement of common maternal mental health problems over the time these data were collected. It is reasonable therefore to attribute the outcome to the intervention.

We acknowledge nevertheless that this study has limitations. Although attrition was low and participation fractions were adequate, the strength of this evidence is limited by potential selection bias because couples were not randomised to intervention or control conditions. There were differences in baseline characteristics which might have influenced the outcomes. Some of these might have been protective of mental health: women in the intervention group were older, were more likely to be in higher status occupations and were more likely to speak English at home and therefore to have easier social participation. Fewer had unintended pregnancies and more had established breastfeeding than women in the control group. However, the babies of women in the intervention group cried and fussed for longer in twenty-four hours than those in the control group, which might have increased the risk of depression in these women [51].

Although differences in a comprehensive set of relevant baseline characteristics were controlled for in analyses, the possibility that people with an unknown, but better adaptive capacity and lower likelihood of developing a common postpartum mental disorder, were recruited to the intervention group remains. However, the well-established determinants of postpartum depression: past psychiatric history, quality of intimate relationship (IBM Care and Control scores and ability to confide in and perceived support from the partner), current mood (EPDS scores and self-rated confidence in infant care) and vulnerable personality characteristics (VPSQ Vulnerability score) did not differ between groups at baseline. In addition, all participants received the "benevolent attention" of participation in detailed structured interviews about matters of direct relevance to their current experiences. Overall, we believe that the potential for bias is unlikely to account for the magnitude of the effect that was found. However, the findings should be interpreted with caution.

It is perhaps unsurprising that a brief (half day) intervention was insufficient to reduce the elevated risk of postpartum mental disorders in women with a history of mood, panic or eating disorders. However, there was very high satisfaction with the program in those who attended the face-to-face session: almost all participants found the knowledge and learning opportunities it provided relevant, timely and valuable. It did not cause harm.

The limited effectiveness for prevention of postnatal mental health problems in women with a past psychiatric history, suggests that a stepped approach in which a universal program is one element in a comprehensive mental health care system might be beneficial. The group with additional needs can be readily identified by primary care professionals by simple questioning, and referred for additional assistance, perhaps including specifically tailored supplementary programs. These could include other psychosocial interventions, for example, structured peer support which has been shown by Dennis et al [67] to be effective in preventing postnatal depression in women identified by screening as being at high risk; and individual consultations with maternal and child health nurses about how to manage infant sleep problems, found by Hiscock et al [68] to lead to significantly lower levels of depressive symptoms.

However, this novel intervention has merit. It appears that the approach is sound, and that offering a salient, acceptable, well-theorised, gender-informed, timely, non-stigmatising, psycho-educational program to couples and their first babies promotes optimal interactions with the benefit of reduced postpartum anxiety, depression and adjustment disorders in the majority of women. It suggests too that our hypothesised mechanisms of seeking to optimise interactions between mother, father and newborn so that empathy and affirmation are increased and criticism, irritability and insensitivity decreased; and to encourage both partners to participate more equally in the increased unpaid workload and infant care were effective.

This intervention is innovative in several ways. First, it includes partners and babies, and focuses on the modification of social risk factors, specifically the quality of day-to-day interactions in a woman's intimate relationships with her partner and her first infant. Second, it recognises that postpartum anxiety and adjustment disorders are common, but less well recognised than depression and require direct attention. Third, it is informed by plausible causal mechanisms that have not been delineated in previous trials. These include: that infant crying and resistance to soothing can arouse anxiety, helplessness and a sense of incompetence; that women experience many unrecognised losses in having a baby; and that disabling occupational fatigue is widespread. Together these can be conceptualised as experiences of entrapment, humiliation and grief which increase potential for depression and anxiety [53]. Rather than positioning men and infants as victims of a woman's mental state, it conceptualises intimate relationships as reciprocal and modifiable. Fourth, the intervention is gender-informed in naming infant care and household tasks as work and making it explicit that failure to recognise the unpaid workload or to share it fairly contributes to occupational fatigue and interpersonal conflict. Finally, rather than just offering support, the intervention was psycho-educational in providing salient knowledge, active learning opportunities and skills training at a critical life-stage. It is framed as a health promotion activity of universal relevance in response to heightened learning needs common to all new parents and is intended not to be stigmatising.

This intervention sought to address possible limitations in the existing prevention trials and distinguished between *de novo *and recurrent mental health problems. It addressed diverse mental health problems including anxiety and adjustment disorders and not just depression. It aimed to modify salient aspects of a woman's intimate social environment, rather than aspects of her individual functioning. It is integrated into primary health care in a local setting and capitalises on an optimal shared learning environment [69].

The study was conducted in seven study sites, which were chosen to achieve diversity in socioeconomic status and in rural and metropolitan locations, and involved systematic recruitment of all couples meeting inclusion criteria. Participation in the study and the intervention appears to have been more appealing to people who were better educated and occupied higher socioeconomic positions. Although the program is intended to be universal, it is unlikely that the face-to-face professionally-facilitated model will reach everyone. This suggests that other modalities might be required to make this knowledge and these skills more widely available to people with lower language skills and emotional literacy. The effectiveness of the intervention was tested when implemented by trained, highly skilled practitioners and it is unknown whether this intervention will be effective when integrated into existing standard primary health care. This novel approach now requires testing in a pragmatic cluster randomised controlled trial.

## Conclusions

A universal, brief psycho-educational group program for English-speaking, first-time parents and babies in primary care appears to reduce de novo common postpartum mental health problems in women. A universal approach supplemented by an additional program may improve effectiveness for women with a psychiatric history.

## Competing interests

The authors declare that they have no competing interests.

## Authors' contributions

JF and HR conceptualised and developed the intervention, obtained funding and conducted the study. JF, HR and KW were involved with the acquisition and management of study data. KW undertook the analysis and all authors were involved in interpretation of the data, with additional expert statistical advice from the Statistical Consulting Centre, University of Melbourne. JF and HR drafted the manuscript with critical revision from KW. JF is the guarantor. All authors have read and approved the final manuscript.

## Pre-publication history

The pre-publication history for this paper can be accessed here:

http://www.biomedcentral.com/1471-2458/10/432/prepub
